# Drug-food Interactions in the Era of Molecular Big Data, Machine Intelligence, and Personalized Health

**DOI:** 10.2174/2212798412666220620104809

**Published:** 2022-01-17

**Authors:** Romy Roy, Shamsudheen Marakkar, Munawar Peringadi Vayalil, Alisha Shahanaz, Athira Panicker Anil, Shameer Kunnathpeedikayil, Ishaan Rawal, Kavya Shetty, Zahrah Shameer, Saraswathi Sathees, Adarsh Pooradan Prasannakumar, Oommen Kaleeckal Mathew, Lakshminarayanan Subramanian, Khader Shameer, Kamlesh K. Yadav

**Affiliations:** 1 Molecular Robotics, Cochin, Kerala, India;; 2 Sanaria Inc, Rockville, MD, USA;; 3 Mar Athanasious College for Advanced Studies, Tiruvalla, India;; 4 Thiruvalla, Kerala; People Care Health LLP Thrissur, Kerala, India;; 5 Urbana Middle School, Urbana, MD 21754, USA;; 6 University of Washington Seattle, Washington WA, USA;; 7 Indian Institute of Information Technology (IIIT), Kottayam, Kerala, India;; 8 Department of Computer Science, Courant Institute of Mathematical Sciences, New York University, New York, NY, USA;; 9 Northwell Health, New York, NY, USA and Faculty of Medicine, Imperial College London, London, UK;; 10 School of Engineering Medicine, and;; 11 Department of Translational Medical Sciences, Center for Genomic and Precision Medicine, Texas A&M University, Houston, TX 77030, USA

**Keywords:** Drug-food interactions, nutrigenomics, precision medicine, machine intelligence, big data, pharmacomicrobiome

## Abstract

The drug-food interaction brings forth changes in the clinical effects of drugs. While favourable interactions bring positive clinical outcomes, unfavourable interactions may lead to toxicity. This article reviews the impact of food intake on drug-food interactions, the clinical effects of drugs, and the effect of drug-food in correlation with diet and precision medicine. Emerging areas in drug-food interactions are the food-genome interface (nutrigenomics) and nutrigenetics. Understanding the molecular basis of food ingredients, including genomic sequencing and pharmacological implications of food molecules, helps to reduce the impact of drug-food interactions. Various strategies are being leveraged to alleviate drug-food interactions; measures including patient engagement, digital health, approaches involving machine intelligence, and big data are a few of them. Furthermore, delineating the molecular communications across diet-microbiome-drug-food-drug interactions in a pharmacomicrobiome framework may also play a vital role in personalized nutrition. Determining nutrient-gene interactions aids in making nutrition deeply personalized and helps mitigate unwanted drug-food interactions, chronic diseases, and adverse events from their onset. Translational bioinformatics approaches could play an essential role in the next generation of drug-food interaction research. In this landscape review, we discuss important tools, databases, and approaches along with key challenges and opportunities in drug-food interaction and its immediate impact on precision medicine.

## 1 INTRODUCTION

**Fig. (1):**
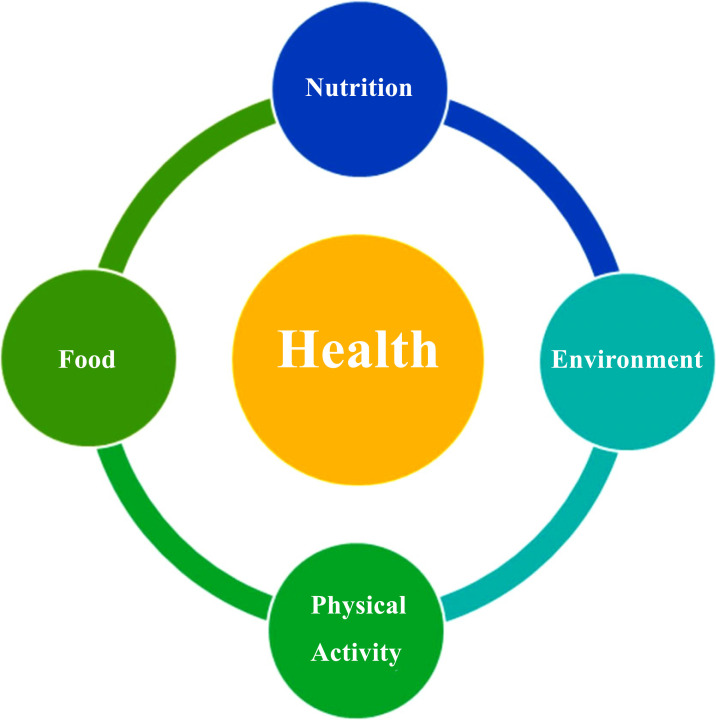
Different aspects that encompass optimal health.

Many medicines contain potent substances that interact inside our system in various ways. Lifestyle and diets have a significant impact on how medications work. A drug interaction eventuates when a substance alters the properties of a drug, causing the effects to be increased or lessened or producing results that neither of the drugs or food could cause on its own. Common types of interaction involve drug-drug interactions and drug-food interactions. These can happen due to unintentional misuse or lack of awareness about the active components in the substances [1-7]. Contrary to the ease with which data on drug-drug interactions is readily available, information on drug-food interactions is often challenging to obtain as humans consume a variety of food that could interact with drugs. Accurately determining the effects of diet and nutrition on a drug is challenging and complex. This article is intended to assist professionals in healthcare and patients in acquiring more knowledge about drug and food interactions. The relationship between food, nutrition, and health is complex and multilayered. Everyone needs food to survive; being healthy is synonymous with survival. The food we eat is one of the vital contributing factors to being healthy (Fig. **1**). Consuming too much food or consuming too little food affects our nutritional level and can result in adverse health consequences; optimal nutrition can be achieved with the right type of food [8]. Translational bioinformatics could play a critical role in understanding the molecular players, interactions, and the impact of food on medicine. FDA categorizes drug interactions into three broad categories (drug-drug interactions, drug-food/ beverage interactions, and drug-disease interactions). The inverse term “food-drug interactions” is also used in the literature to define the same concept. However, in this review, we focus exclusively on drug-food interactions; other interaction themes are out of scope.

## 2 WHAT IS FOOD?

Food is any substance when ingested and digested satiates hunger by providing nutrition without any immediately noticeable harm [9]. The food we consume sustains life by supplying energy *via* nutrients and prompts the organism's growth. Plants and animals are two principal sources of food; while each source has its distinctive merits and demerits, it is beneficial to consume food from both sources than relying on a single source. The food can be divided into four groups as defined below: healthy food, medical food, diet food, and functional food.

### 2.1 Healthy Food

Healthy foods are broad classes of food that supply the body with vital nutrition and adequate calories. Although healthy foods can be obtained from animal and plant sources, they are predominantly plant-based whole food with little to no processing. Eating healthy food complemented by a healthy lifestyle decreases the risk of many chronic diseases [10].

### 2.2 Medical Food

Medical food comes under distinct categories intended and formulated for unique disease management, metabolic deficiencies elicited from scientific principles and medical assessment. For example, Axona and Souvenaid are two medical foods for Alzheimer's disease and memory impairment. Axona provides neurons with an alternative source of energy to glucose, the β-hydroxybutyrate ketone. The randomized phase II clinical trial showed significant improvements in cognitive performance. Souvenaid contains precursors and supporting nutrients to enhance membrane and synaptic formation and function in Alzheimer's disease. Data from a randomized clinical trial showed improvement in the function of verbal recall [11].

### 2.3 Diet Food

Any food whose recipes are reformulated/restructured to reduce fat, carbohydrates, or sugar, making it part of a diet for a specific function like slimming or bodybuilding, is called diet food [12].

### 2.4 Functional Food

Functional food is natural or processed foods containing known or unknown biologically active compounds, providing a scientifically validated and recorded health benefit for the prevention, management, or treatment of chronic diseases in specified, safe, non-toxic quantities [13].

## 3 WHAT IS MEDICINE?

Any synthetic or organic substance that aids in prevention, diagnosis, treatment, healing, and health promotion is medicine. Since ancient times, food has played a vital role in preventing and treating diseases. Hippocrates, the father of modern medicine, once said, “Let food be your best medicine and your best medicine be your food” [14].

### 3.1 Food as Medicine

Since time immemorial, the role of nutritious and healthy food in maintaining health has been well acknowledged. Recent studies show that food can prevent chronic diseases and metabolic syndromes like obesity, diabetes, hypertension, and dyslipidemia [14, 15]. Dietary risk factors were responsible for 11 million deaths and 255 million disability-adjusted life years in 2017. Systematic evaluation of dietary consumption patterns provides a comprehensive picture of the health effects of poor dietary habits. Dietary risks affect people regardless of age, sex, and other sociodemographic characteristics. Improvement of diet could potentially prevent one in every five deaths globally.

### 3.2 Food and Disease

Food availability and individualized diet play a critical role in defining long-term health outcomes. Here we briefly discuss the role of food in the setting of three crucial diseases.

#### 3.2.1 Cardiovascular Diseases

Cardiovascular disease is ranked as the number one cause of death worldwide [15]. The high prevalence of cardiovascular disease in this century can be undeniably linked to poor food choices and unhealthy lifestyles. Disproportionate consumption of nutritionally low but calorically dense foods results in systemic inflammation, changes in insulin sensitivity, poor glucose regulation, and metabolic abnormalities [16]. Consumption of functional food that has cardioprotective properties, such as reducing homocysteine levels, lipid-lowering effects, and antioxidant ability integrated with lifestyle changes, can be widely utilized to prevent cardiovascular diseases (Table **1**) [17].

#### 3.2.2 Diabetes

Diabetes Mellitus type 2 is one of the fast-growing public health problems globally; the relative treatment difficulty makes it expensive to manage. The food we consume plays a crucial role in preventing and managing diabetes. Functional foods, especially derived from plants containing flavonoids, polyphenols, sterols, unsaturated fatty acids, alkaloids, terpenoids, and sterols, can play a vital role in preventing and managing type 2 diabetes. Studies have shown an inverse relationship between plant-based food and the occurrence of type 2 diabetes [23, 24]. The phenolics component, a secondary metabolite of plants, has been found to play a role in preventing chronic diseases, such as type 2 diabetes. The mechanism is thought to be robust, as the phenolic molecule present aids in the suppression of oxidative stress and inflammation. The phenolic compounds also activate the erythroid 2-related factor (Nrf2) pathway, which increases the expression of ARE (antioxidant response element), resulting in increased concentrations of free radical scavengers. Polyphenols also influence carbohydrate metabolism, insulin sensitivity, and inflammation mediators [25-29].

#### 3.2.3 Cancer

A staggering 30% of all cancer incidence can be directly or indirectly linked to nutrition and food [30-33]. Several naturally occurring compounds in plants or plant extracts have exhibited anti-cancerous properties and their ability as a chemo-preventive factor. The root stimulus for cancer is DNA damage, especially by free radicals. Functional foods have proven to reduce oxidative damage and oxidant stress cell division, which is directly associated with mutagenesis and carcinogenesis (Table **2**) [34-44]^.^

## 4 BIOAVAILABILITY, BIO-ACCESSIBILITY, AND BIOACTIVITY

The bioavailability of a nutrient is the percentage of nutrients absorbed through the diet and used for normal body function. The bioavailability of nutrients is associated with both external and internal factors. The external factors include the food composition and the structure of the nutrient. Age, gender, *etc*., constitute the internal factors [45]. The essential step in making a nutrient bioavailable is to emancipate the nutrient from its food matrix and transform it into the chemical form that can bind. The existence of nutrients/minerals in different chemical structures can influence their bioavailability. Dietary iron exists in two forms, haem-iron and non-haem forms of iron. The iron from the haem-iron form is readily and easily absorbed by our body, contrary to the non-haem form, as the haem acts as a protective ring around the iron atom once released from the food matrix. The haem iron form is exclusively found from animal sources, whereas non-haem iron can be obtained from animal and plant sources [46, 47].

**Fig. (2):**
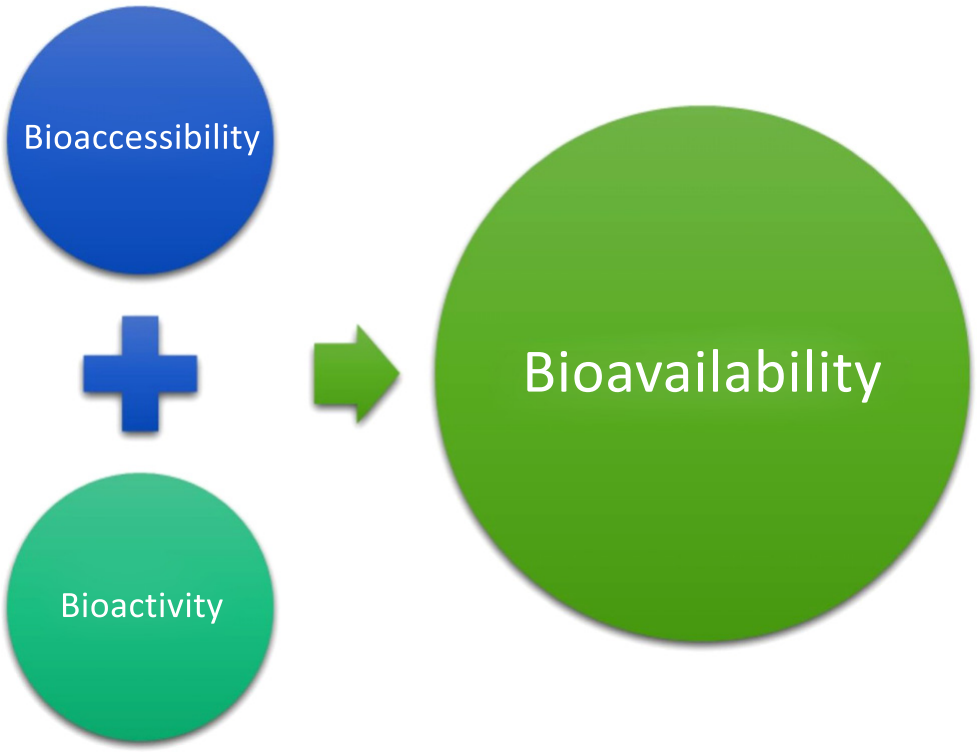
Interrelation between bioavailability, bioaccessibility, and bioactivity.

Bioavailability is interrelated with two other terms, bioaccessibility and bioactivity (Fig. **2**). Bioaccessibility is the number of nutrients liberated from the food matrix, detectable in the gut, and passing through the intestinal barrier. Bioactivity is the effect brought upon by the nutrients that exert changes in health status. Bioactivity measurement is rooted in events occurring when a bioactive compound interacts with the biomolecules; these interactions set off a metabolite /signal or a response that modulates and intensifies until the health benefit is achieved [48]. The bioavailability of nutrients can be enhanced or inhibited. At the absorption site, nutrients can either interact with each other or with other dietary components or biomolecules, resulting in a change of bioavailability. An enhancer increases bioavailability, whereas the inhibitor decreases bioavailability. For example, adding fat/ oil to the meal improves the bioavailability of carotenoids, as they are fat-soluble. Vitamin C enhances the absorption of iron 2-3 times [49]. The medicinal properties of curcumin, an active compound in turmeric, cannot be effectively utilized because of its extremely low bioavailability. Piperine, an alkaloid present in black pepper and long pepper, alters the pharmacokinetics of curcumin. The concomitant administration of piperine (20mg) increased the bioavailability of curcumin by 2000%. The study revealed piperine enhanced serum concentration, absorption, and bioavailability [50].

## 5 DRUG-FOOD INTERACTION

Drug-food interactions are characterized as changes in the pharmacokinetics or pharmacodynamics of a drug or nutritional element and a reduction in nutritional status. Not all drugs interact with food items. A particular type of food can trigger a range of interactions with drug mechanisms. These problems may range from slowing down the possible drug effects to causing adverse effects on the body [1]. For instance, the significant fluid volume would reduce the gastric emptying rate of drugs ingested in specific forms of human physical activity. Similarly, a large amount of solid food consumption may reduce the gastric emptying rate of drugs consumed [51]. The components of foods may directly impact the pharmacokinetics process in several ways. The solutions and compounds of the drug will be affected by food consumption, and the absorption may be delayed for several hours [52].

### 5.1 Drug-food Interactions in a Clinical Setting

Drug-food interactions significantly influence the efficacy of drug treatment and the side-effect profiles of drugs. The clinical care team must be well educated and aware of the relationships and interactions between foods, their constituent nutrients, and drugs for successful drug treatment. When drugs enter the human body, they may cause drug-induced conditions; some of the states are fatal and life-threatening [53]. They pose severe risks to patients when administered together with food. When Ciprofloxacin, an antibiotic commonly prescribed to treat respiratory tract infections, is applied concurrently with calcium-fortified orange juice, it reduces the bioavailability to 40% over time. It may harm patients with adverse outcomes, such as the development of antibiotic resistance. Additionally, when ciprofloxacin is taken with dietary molecules contained in milk, it lowers the bioavailability and reduces the dissolution efficiency compared to the consumption of ciprofloxacin with drinking water [6].

### 5.2 Drug-food Interaction Types

**Fig. (3):**
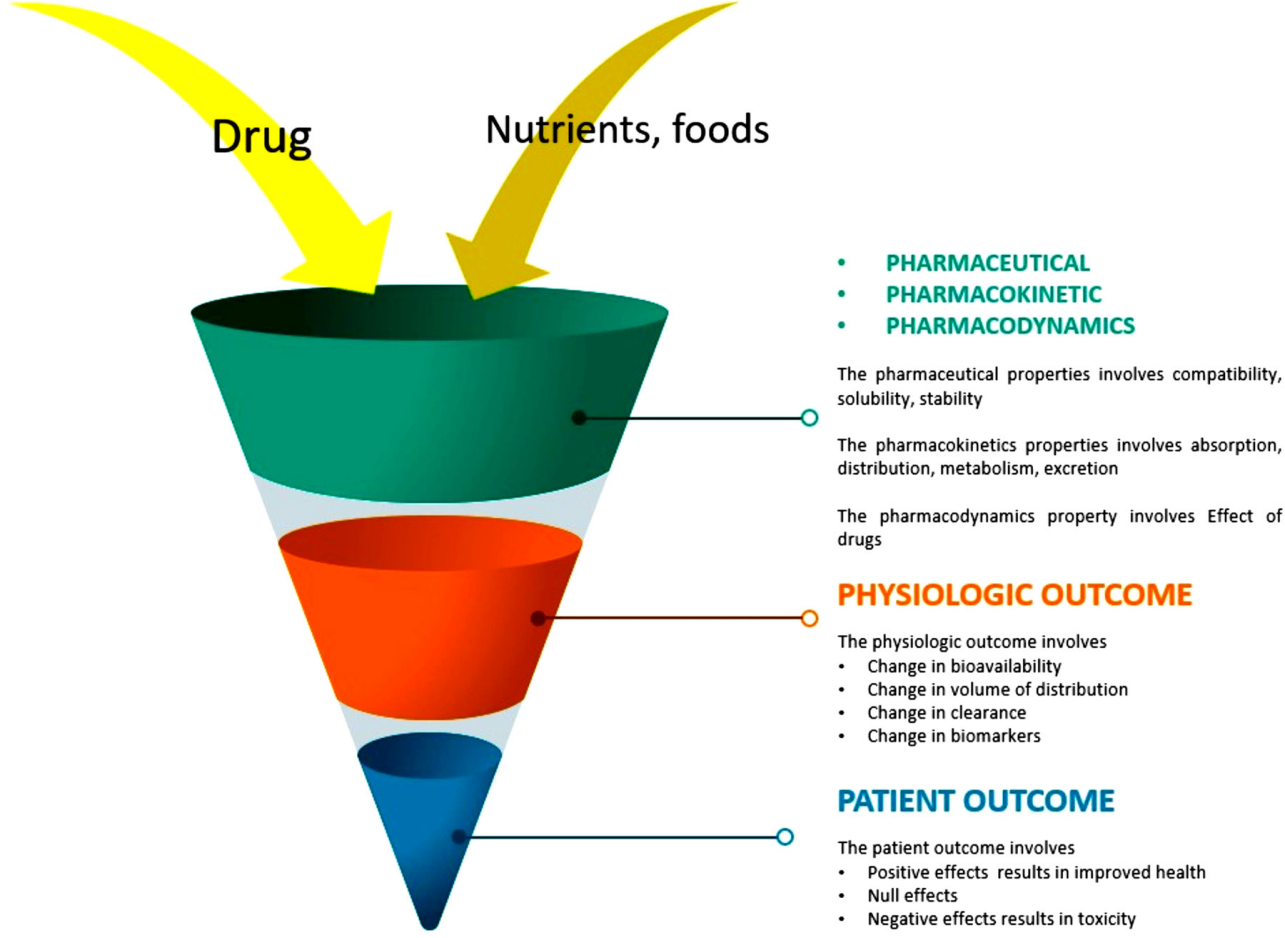
Possible physical, chemical and kinetic changes due to drug-nutrient food interaction.

The scaling of drug-food interaction studies in its nascent stage is limited to ascertaining whether a meal would interfere with the bioavailability of a drug. Recent research studies affirmed that drug-nutrient interactions could alter the pharmacokinetics and pharmacodynamics of drugs (Fig. **3**) [54, 55]^.^ Pharmacokinetics is the quantitative elucidation of drug dispositions that consists of absorption, metabolism, distribution, and excretion. In contrast, pharmacodynamics refers to the clinical effects, which can be either a decrease in bioavailability or an increase in bioavailability, both of which can result in toxicities [54, 55]. There are mainly four types of drug-nutrient interaction: Type I, Type II, Type III and Type IV.

#### 5.2.1 Type I

Type I interactions happen even before the entry of drugs and nutrients inside the body. It mainly occurs during the preparation formulation or administration process where the interacting elements directly contact each other. Patients receiving parenteral or enteral nutrition are at greater risk of type I interaction. Type I interaction is visible when you mix drugs with food types. The drug Levothyroxine can interact during enteral nutrition, causing poor dissolution and possible adsorption to the tubing, leading to hypothyroidism [56, 57].

#### 5.2.2 Type II

Type II is the most common type of interaction, which occurs mainly when drugs and nutrients are administered orally or enterally. Type II can be further classified into Type A, Type B, and Type C.

##### 5.2.2.1 Type A

In this interaction, the enzymatic function is modified. The altered enzymatic function increases the bioavailability and may lead to toxicity. The cyclosporine and grape juice interaction is a classic example of type A interaction. The interaction inhibits intestinal CYP34A, causing increased bioavailability of cyclosporine. Clinically, the increase in the concentration of cyclosporine leads to toxicity [56, 57].

##### 5.2.2.2 Type B

Type B interactions are characterized by an altered transport protein function solely responsible for the transport of agents before entering the systemic circulation, resulting in malabsorption of dietary components. The interaction of valproic acid found in berries and L-carnitine leads to competitive inhibition of the transport protein SLC22A resulting in malabsorption of dietary carnitine. [56, 57]

##### 5.2.2.3 Type C

Type C interaction is characterized by chelation, binding, or complexation of drug and dietary components, leading to deactivation or reduced bioavailability. Ciprofloxacin and calcium supplements form complexes of Ciprofloxacin with calcium ions resulting in reduced bioavailability of Ciprofloxacin [56, 57].

#### 5.2.3 Type III

Type III interactions involve changing the volume of distribution of cells or tissues, changes in metabolism, and penetration to specific tissues or organs. Type III interactions can be reduced by adjusting the dosage to the optimum amount. The interaction of rasagiline and tyramine in large amounts causes an acute hypertensive attack referred to as cheese reaction [56, 57].

#### 5.2.4 Type IV

Tissue-specific Type IV interaction occurs when drugs and nutrients compete to eliminate transporter proteins. Modulation, antagonism, and enterohepatic elimination are some of the consequences of Type IV interaction [56, 57].

### 5.3 Food-drug Interaction: Experimental Studies

Drug-food interactions may have two major clinical effects: decreased bioavailability of a drug, which raises the likelihood of adverse effects, or increased bioavailability, which predisposes to treatment failure [58]. Bioavailability is an important pharmacokinetic parameter correlated with the clinical outcome of most drugs [59]. Drug transporters and drug-metabolizing enzymes play essential roles in drug ingestion, delivery, digestion, and elimination. The pharmacokinetics and pharmacodynamics of medication may be affected by individual or combined actions. One of the confounding factors that have recently been shown to lead to possible complex drug interactions is the interaction between drug-metabolizing enzymes and transponders [60].

#### 5.3.1 Food and Drug Transponders

Drug transponders in the gut and liver regulate absorption and bioavailability, which is critical in deciding oral drug disposition. Many secondary metabolites known as phytochemicals are present in everyday foods like fruits and vegetables, and many have been linked to health benefits. Recent studies show that some of the phytochemicals are substrates and modulators of specific members of the superfamily of ABC transporting proteins [60].

#### 5.3.2 Food and Metabolizing Enzymes

In general, drug metabolism reactions are divided into two stages. Phase I reactions (oxidation, reduction, and hydrolysis) are mainly regulated by the cytochrome P450 (CYP) family of enzymes. Phase II reactions combine the drug or its phase I-derived metabolite with an endogenous compound, such as glucuronic acid, glutathione, or sulphate, to create a more polar end-product that can be excreted more easily (Tables **3** and **4**) [61-70].

#### 5.3.3 Drug-food Interaction Network

**Fig. (4):**
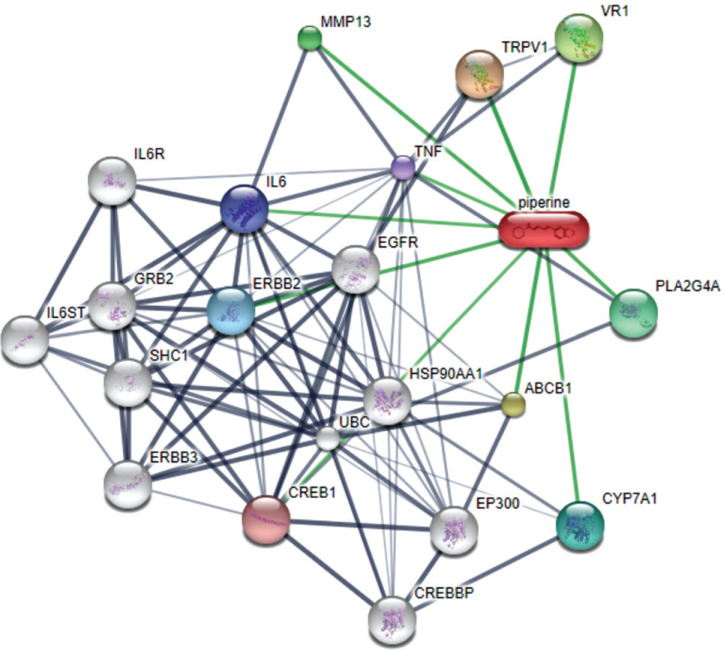
A network graph of Piperine the active component of black pepper. Stronger associations are represented by thicker lines. Protein-protein interactions are shown in grey, chemical-protein interactions in green and interactions between chemicals in red.

Knowledge of drug-food interactions aids to identify, predict, and to prevent unfavourable interactions between food and marketed or novel drugs [71]. To interpret the biological responses to diet, as well as to assign the importance of drug-food interaction, we need to assemble all available information on the chemical background of the small molecules in our diet. “NutriChem” is such a database that helps us estimate the effect of plant-based food on health based on the availability of information related to the food's small molecule constituents [72]. Integrating the “NutriChem” biological activity data of marketed drugs makes it possible to study the effect of diet on drug properties related to their pharmacokinetics and pharmacodynamics, and create drug-food interaction networks that help the patients and clinicians easily understand the food items that must be potentially avoided while under medication (Fig. **4**) [72, 73].

The drug-food interaction network allows system-level analysis in revealing the underlying mechanism behind the drug-food interactions. A network is simply a graph with nodes and edges. Nodes are entities that we evaluate, and edges are the connections between them. Edges convey information about the linkage between the nodes [73]. We can systematically visualize the interaction and its properties by retrieving interaction data from “NutriChem” and constructing different drug-food interaction networks. Drug-food-target protein interaction networks are based on unique protein interactions shared between different foods, and they can be made in different ways. One is a network of foods as nodes that interact with the same drug target proteins.

The node size reflects the number of phytochemicals, and the edge width represents the number of interacting proteins common to the two foods. Another way is by considering drug targets as nodes and connecting them when a minimum number of foods having active interaction for both the drug targets are found. Although a given food can have activity for drug targets of various disease categories, creating an individual network for each disease category is convenient [71-73]. Networks are also created based on drug-food interactions affecting pharmacodynamics or pharmacokinetics. Phytochemicals in the diet interact with proteins within enzymes, transporters, and carriers other than drug targets. These networks have drug targets or proteins within the rest of the categories (enzymes, transporters, or carriers) as nodes along with drugs and food are complex with multiple factors considered at a time. It can be effectively dealt with by assigning different shapes to different nodes (circle= protein target, triangle= drug, diamond=food) and various edge colors for different phytochemicals. This helps in understanding the activity of food more closely. Incorporating more factors into the drug-food interaction network helps to understand it better. One such factor yet to be considered is the amount of food and drugs. It is of much relevance as the bioavailability of the drug has a major role in the pharmacokinetic activity. Right drugs, along with the right food in the right amount, are what we need. Quantified drug-food interaction networks can be considered as the key to this [71-74].

### 5.4 Medicines Exhibiting Poor Bioavailability in the Presence of Food

Drug-food interactions can recklessly reduce or increase the drug's impact. Some commonly used herbs, fruits, and alcohol may cause treatment failure to a point affecting the patient's health. Most clinically significant drug-food interactions are triggered by changes in the drug's bioavailability caused by food (Table **5**) [75-80].

#### 5.4.1 Pharmacomicrobiome: The Complex Relationship between Drugs and the Microbiome

Gut microbiota may play a primary role in human health by affecting the immune response, intestinal homeostasis, nutrient processing, energy harvesting, and pathogen resistance. An increase in the risk of various diseases, such as obesity, inflammatory bowel disease (IBD), cancer, diabetes, psychiatric disorders, and asthma has been related to an imbalance of the gut microbiota [81]. Many studies have proposed that the mechanism is bidirectional, suggesting that the microbiome affects the drug and the microbiome. This hypothesis was tested in a clinical trial. Statins reduce circulating LDL by promoting the growth of gut bacteria that produce bile salt hydrolases, enzymes that break down the bile acids to digest fatty foods. Gut microbes may contribute to drug efficacy and protection by enzymatically modifying drug structure and altering drug bioavailability, bioactivity, or toxicity [82]. Proton pump inhibitors, selective serotonin reuptake inhibitors, and laxatives are drugs that affect the composition and function of the gut microbiome. Changes in the gut microbiome caused by proton pump inhibitors can decrease colonization resistance and the development of enteric infections, such as *Clostridium difficile* infections [83]. Gut microbiome composition is associated with antitumor response and the clinical efficacy of treatment with immune checkpoint inhibition [81]. Understanding how microbes and drugs interact may lead to new therapies or improvements in administering current medications. For example, physicians may be able to predict how a patient might respond to a drug based on their gut bacteria and modify the patient's dosage accordingly. Dietary changes or antibiotics might also be recommended to make a person's gut microbiome more receptive to a drug.

## 6 STRATEGIES TO TRACK, MONITOR, AND ALLEVIATE DRUG-FOOD INTERACTIONS

One of the primary barriers to avoiding drug-food interaction is the lack of readily available information. While FDA and other regulatory agencies actively monitor such interactions and include leaflet information as part of medication dispensing. The patients may not routinely refer to such information. Innovative approaches are now required to remind patient communities of the potential risk of drug-food interactions routinely. Resources like DrugBank (See: https://go.drugbank.com/drug-interaction-checker) and other websites provide interaction checkers [84]. In the following section, we discuss key transdisciplinary approaches.

### 6.1 The Emerging Role of Digital Health in Patient-reported Outcomes in the Setting of Drug-food Interaction

Digital health refers to the use of information technology, communication technologies, and electronic technologies for better healthcare delivery [85]. Digital health ameliorates computational technologies, smart devices, computational analysis techniques, and communication media to help healthcare professionals and patients. The inception and the rapid growth of digital health can be seen as parallel with the digital revolution because all enabling technologies of digital health are the result of the digital revolution, such as mass production of digital circuits, microfabricated devices, novel communication technologies, cellular phones, computers, and even the internet. It started in 1897 with telemedicine when a child was diagnosed with croup cough *via* the telephone [86]. It was the first remote diagnosis reported. The introduction of the edge notched cards of the early nineties, Hollerith punched card for electromagnetic data tabulation and storage, completion, and installment of ENIAC (Electronic Numerical Integrator and Computer), the first general-purpose digital computer at the University of Pennsylvania, led to the development of the timeline [87]. Later, the rapid growth of digital health parallels technological milestones, such as the invention of the point-contact transistor, the first type of transistor to be successfully demonstrated, at the Bell Laboratories in 1947, *etc*. [88]. Other milestones of digital health were the first implantable cardiac pacemaker which itself was enabled by the development of the transistor, the release of the first Holter monitor (cardiac activity monitor) in 1962 [89], the proposal of relational database by E. F Codd in 1970 that set the stage for large datasets, the introduction of the first automated blood analyzer by Technicon in 1974, the first clinical CT scanner in 1975, the introduction of the first generation LIMS (Laboratory Information Management System) and the introduction of user-friendly infusion pumps in 1982, the introduction of the first wearable consumer wireless heart rate monitor in 1983, the introduction of the first commercial automated DNA sequencer in 1987 [90], home glucose meter in 1987 which was one of the first commercial bio-sensors, the introduction of the compact automatic wrist cuff blood pressure monitor by Panasonic in 1993, the first chip-based DNA sequencing technology in 1996, the launching of the Da Vinci robotic surgical system and the Publication of the “draft” map of the Human Genome in 2000 [91]. Moreover, the formation of organizations, like Healthcare Information and Management Systems Society (HiMSS) in 1961, Health Level Seven International in 1987, American Medical Informatics Association in 1989, Documentum content management platform in 1990, The LOINC (Logical Observation Identifiers Names and Codes) database in 1994, and the Clinical Data Interchange Standards Consortium(CDISC) in 1997, was game-changing [92]. The elements of digital health include wireless devices, sensor varieties, advanced software technologies, the internet, social networking, and other long and short-range networks. And these technologies have helped the advancement and development of prognosis, diagnosis, and treatment methods. Digital health approaches in prognosis facilitated unprecedented growth in recent times. The availability of large databanks or databases, the popularity, and advancement of Electronic Health Records (EHR), and clinical data systems help predict the future development of a disease, expectation of the quality of life, probability of health issues, and complications and likelihood of survival. Along with these technologies, advanced sensors to collect data, wearable devices to monitor health status, and advanced algorithmic techniques like statistics-based algorithms and neural network-based algorithms helped forecast diseases and health conditions [93]. Digital tools are used in diagnosis, such as smartphone-based scanning devices, portable and highly personalized diagnostic devices leveraging nanotechnology and MEMS (Micro Electro Mechanical Systems), cheap and advanced microfluidics systems like Lab on a Chip (LoC) for rapid testing of body fluids. Recent innovations including novel micro designs of diagnostic tools, biocompatible and invasive sensors, ultra-thin and low power chips and transistors, advanced low power, and high-efficiency sensors, and nanotechnology-based point-of-care diagnosis are adding fuel to the future of digital health [94, 95]. These technologies help medical and healthcare professionals make better informed decisions and facilitate the prevention and early diagnosis of life-threatening diseases. From mobile medical applications and software systems to artificial intelligence and machine learning algorithms, digital technology has been a driving revolution in healthcare. This convergence of people, information, technology, and connectivity will change how we deal with prognosis, diagnosis, treatment, and healthcare delivery.

#### 6.1.1 Leveraging Social Media Platforms to Improve Drug-food Interaction Awareness and Monitoring

Social media plays a vital role in behavioural psychology today. 3.8 billion people (that is 50% of the global population) use social media as of Jan 2020, according to Hoot suite social media statistics [96]. These digital platforms could spread the need and importance of adhering to medication administration instructions. According to the Hoot suite statistics, users spend around 2 hours per day on social media. Many actions taken by people are based on their engagement in these platforms. Therefore, different stakeholders can communicate general awareness of drug-food interaction through these platforms. Patients can also be provided with options to clarify their questions on the social media pages of pharmacies. Pharmacy services can create loyal patient customers and add value to their business profile [96].

### 6.2 Drug-food Interaction Platform for Checking Potential Drug-food Interactions

A reliable and user-friendly app to check potential interactions with drugs will help pharmacists ensure vital drug information communication while filling prescriptions. Even patients themselves could also check for any possible drug/food interaction and seek appropriate medical advice from their physician or pharmacists. We list the three most reliable and user-friendly apps here. Furthermore, we discuss the design and system architecture of a wearable device and companion app that could help track eating habits and potential drug-food interaction patterns.

#### 6.2.1 Drugs.com

Drugs.com is a multipurpose platform for drug information. It hosts the disease, drug, FDA alert, drug comparison, pill identifier, and treatment guide. It provides provisions for Drug/Drug, Drug/Food, and Drug/Disease interaction. You can check all interactions concerning drugs by typing the drug name in the search box, unlike other platforms where you have to write the comparison drug or food to check whether they interact. It is available on iOS and Android platforms with a 4.4 and 4.7 rating, respectively. It does not require any subscription like IBM, Micromedex or Lexicomp. Drug.com is Health On Net (HONcode) certified [97].

#### 6.2.2 Medscape

Medscape is one of the widely used platforms by medical professionals to search for drug and disease-related information. It provides in-depth drug information, including dosing and pharmacokinetic parameters. It also has provisions for the latest medical news, CME, and other medical-related courses. The drug interaction checker option in Medscape gives different levels of interaction from “Serious: Use alternative,” “Monitor closely,” and “Minor.” Food/herbal interaction with grapefruit, green tea, garlic, ginger, *etc*., can be checked in Medscape. It is also available both on iOS and Android with a rating of 4.3 and 4.6, respectively [98].

#### 6.2.3 WebMD

WebMD is another well-known online medical news and information platform, especially for consumers. It is accredited by Utilization Review Accreditation Commission (URAC) and HONcode. The drug interaction checker option on WebMD provides four types of interactions somewhat similar to Medscape as 1. Do not use together, 2. Serious 3. Monitor Closely and 4. Minor. A minimum of two compared drugs/food has to be entered to get the interaction information, unlike Drugs.com. It is available on iOS and android with a rating of 4.6 and 4.5, respectively [99].

#### 6.2.4 BH Glove: Monitoring Drug-food Interaction using a Wearable Device-app Integration

While there are multiple wearable devices, apps, and monitoring services available to monitor food intake and get a quantified view of an individual’s calorie intake, no device is available, to the best of our knowledge, that can passively monitor food intake and alert the patient. In this context, a new device model, a smart connected glove that can monitor mechano-sensing of eating patterns, is proposed. The BioHeart glove (BH Glove) is an open-filtered glove synced with an app and integrates monitoring of multiple physiological parameters, including heart rate, oxygen level, and blood pressure. Furthermore, the app integrated with the glove can remind patients to take medications, and it notifies when patients need to condition. It tracks blood pressure using data, as well as using unique trackers embedded in each finger. The blood pressure level is shape coded, and based on variability in blood pressure, the patient gets a notification on your phone from the app. The sensor on the back of your hand can provide visual or voice indicators (lighting up or making a chiming noise) to remind the patient to sleep passively or actively, drink more water, exercise, or take their medicine. It can also intelligently track the motions of eating certain things (junk food, vegetables, *etc*.) and tell the patient when to stop, or it can chime, reminding the patient not to give in to the cravings. Patients can get healthier recipes from the app and other alternatives on a companion phone application or web application. The app uses data from the glove and personal preferences to make things such as a meal plan, a medication schedule, exercise routine, and other things. Patients also would get reminded that they cannot eat certain foods or bring their pressure higher or lower. The companion app can also track medicine, recognize food, retrieve content, and discuss potential drug-food interactions.

### 6.3 Shared Decision Making

The patient-centric approach is vital to achieving the desired therapeutic outcome, and thus it is a fundamental pillar of clinical practice. A right therapeutic plan must be framed based on patient preference coupled with evidence-based practice to ensure patient rights on ethical grounds [100]. Physicians should seek an alternative based on the patient's diet habits if those options can also lead to the desired outcome to a great extent. Providing different treatment options and asking patients' preferences will help improve patient adherence to therapy and proactive involvement. Moreover, patients are better informed in today's Google era than they were a few decades earlier. They appreciate if their concerns and preferences are considered in selecting appropriate treatment regimens. Such a shared decision model will help alleviate the untoward interaction of drugs with food or the patient's other nutrient intake [101].

## 7 ROLE OF MACHINE INTELLIGENCE AND BIG DATA IN PRECISION HEALTH

Machine intelligence and other artificial intelligence-based technologies have helped the medical industry grow to an unprecedented clinical and diagnostic advancement level. Big data and machine learning have contributed mainly to prognosis and diagnosis [102]. Accurate forecasting of diseases and conditions with time series prediction algorithms and speedy diagnosis from image scanning using advanced computer vision algorithms are some examples [103]. Application-level Artificial Intelligence algorithms are of two types, statistical mathematics-based algorithms called machine learning algorithms and bioinspired neural networked algorithms called deep learning algorithms. Traditional statistical methods and techniques enable easy handling, analysis, processing, and decision making, using time series data like clinical data. Statistical methods, such as ARIMA, SARIMA, *etc*., were used earlier, and now most of these prediction algorithms are replaced by efficient and better-performing deep learning-based algorithms and techniques [104]. Deep learning algorithms are designed to deal with time-series data; data with time-based relations are called recurrent algorithms. A first of its kind is the Recurrent Neural Network (RNN) that can process and make predictions on time series data, such as clinical data of lab measurements and DNA sequences. Advanced models of RNN such as LSTM (Long Short-Term Memory) and GRU (Gated Recurrent Unit) have been helping computational biologists and bioinformatics engineers to make more accurate and fast predictions on medical time series data [105]. This time series analysis and forecasting process mainly helps in scenarios, such as cardiac arrest predictions in the ICU and patients' ECG records [104]. Big data and large medical databases also play an important role. Big data is a vast quantity of data that may be of any sort, such as health records, which can be processed and analyzed by supporting technologies. The most exciting feature of big data in healthcare is that it can provide more precise and personalized care. Big data analysis gives a detailed picture of patients and even large populations with which healthcare practitioners can determine how a particular patient will respond to a specific treatment or even identify at-risk patients before a health issue arises [106].

One of the main applications of big data is in predictive medicine and population health management. It helps in patient prediction from population data and helps in better staffing and staff shift management in ICUs, especially in emergencies like a pandemic outbreak. Another application of big data is on the HER (Electronic Health Records), where every patient will have their digital record, including demographics, medical history, allergies, laboratory test results, *etc*. This can be used for analysis and forecasting [107]. To support this, several databases are also present, such as the MIMIC critical care database. Another set of algorithms and techniques are computer vision-based algorithms that specifically deal with images and video. The digital health scenario that deals with medical images and videos has revolutionized the areas, like medical scanning and diagnosis. It all started with the use of deep neural networks for visual applications, such as image classification and recognition. Kunihiko Fukushima, in 1979, developed a dedicated network for this called neocognitron [108]. This was followed by several visual-based algorithms, such as CNN (Convolutional Neural Network), which is an inspiration from the human brain's visual cortex, and LeNet by Yann LeCun in 1998 [109].

These algorithms in the deep learning timeline have been used in digital health and diagnosis, mainly in computer-vision-based applications, such as image segmentation and classification. Besides using mainstream deep learning algorithms and techniques, researchers also developed dedicated algorithms for medical images and videos [109, 110]. The U-Net is an example; U-net comes from the autoencoder family of deep learning algorithms and was developed specifically for the segmentation of bio-medical images such as brain scan images. It made the process of dealing with multi-modal scan images easier, which was a complicated process earlier. It helped in speedy segmentation of brain parts such as tumorous areas and helped in pathological diagnosis [111]. After this, many techniques were introduced exclusively for biomedical applications, like 3D U-Net and WNet. In the COVID-19 pandemic outbreak, these two subareas of deep learning have helped clinicians and healthcare professionals worldwide, including prediction, contact tracing of the viral infection, and diagnosis. Many international airports set up passenger screening to identify people with elevated temperature levels, which is a primary symptom of the disease. Many of the screening systems include a thermal imaging camera along with a dedicated deep learning-based algorithm to detect people efficiently [112].

Interaction between drugs and food includes but does not restrict to the well-established domain of protein-protein modeling. Hence, protein-protein interaction prediction models can serve as the backbone for developing DFI (drug-food constituent interactions) models [113]. The most common and regarded strategy, which has also been extended to DFI, is Deep Learning. Deep Learning is a specialization of Machine Learning (and hence, Artificial Intelligence) that uses an architecture of neural networks, which automatically extract the relevant features and predict the interaction outcome. Features can be loosely thought of as (ideally) a minimal representation of the input used to train the network. Since the Deep Learning paradigm is centered on automatic identification and extraction of input features, it is generally preferred over Machine Learning, which requires feature engineering. Deep DDI (drug-drug interaction) is one of the proposed models to predict Drug-Food Constituent Interaction. The Deep DDI model is a multiclass classification network that predicts the probabilities corresponding to each of the 86 labels [114, 115]. However promising Deep DDI may be, it still falls as a victim of the availability of DFI datasets. Recently, Graph Embedding methods have shown to be equally or more effective than the traditional neural networks approach and are a field of interest, attracting many researchers [116]. While it is important to develop tools to predict drug-food interactions, it is equally important to find approaches for incorporating the DFI information in the existing flow of information. Accessibility of information and scalability of the information architecture are important. With the rise of the digitization of healthcare, the mass flow of information in medical scenarios and its maintenance has become easier. Integrating the existing DFI database with digitized medical records can help the nonmedical population be educated about their dietary restrictions. It would be easy to access, update, and scalable. Another interesting approach would be to display the DFI information interactively with the existing virtual reality technology. A smartphone with internet connectivity can scan images using its camera and search the interaction of the scanned foods with the pre-fed drugs being medicated. In the future, it can also be integrated with wearable technologies, like wearable contact lenses or glasses, most of which are currently in their developmental stages. However, security and encryption concerns, compatibility issues, and lack of acceptance of the technology are a roadblock to the development [116, 117].

## 8 PERSONALIZED WELLNESS: NUTRIGENOMICS AND NUTRIGENETICS

Nutrition research is at its golden peak, addressing and finding answers to the future of human health through diet and nutrition. Each individual is unique in terms of their genetic makeup; genetic variation makes them respond to different nutrients/food/diets differently. The research of nutrigenomics and nutrigenetics considers the genome genomic variations and makes nutrition deeply personalized. Nutrigenetics and nutrigenomics are the effects of the genome and genetic variations on food/nutrients intake and its role in gene expression. High-throughout' omic' technology, along with genomic information, allows us to understand the nutrient-gene interaction and helps in developing personalized nutrition [118].

### 8.1 Nutrient Gene Interactions

The nutrients in our body can interact with our genes. Fundamentally, the nutrient-gene interactions can occur in three ways and could potentially act as drivers of drug-food interactions (Table **6**) [119, 120].

#### 8.1.1 Carbohydrate and Gene Expression

High intake of carbohydrates stimulates the transcription of genes that encode the following enzymes: glucokinase (GK) and pyruvate kinase for glycolysis, ATP citrate lyase acetyl CoA carboxylase, fatty acid synthase (FASN), and stearoyl-CoA desaturase 1 (SCD1) for lipogenesis and glucose 6-phosphate dehydrogenase for the pentose pathway, thereby promoting the accumulation of sugar as triglycerides (TGs) [121-126]. Carbohydrate-responsive element-binding protein (ChREBP) is the key protein that governs the transcription of these genes. ChREBP forms a heterodimer with Mlx (Max-like protein X, a member of the c-Myc family of transcription factors) and binds to fructose and glucose, triggering the transcription of target genes. This regulation plays a key role in global glucose homeostasis and sugar-induced lipogenesis. Receptor members of the family Hepatic nuclear factor 4 (HNF-4), liver-X-receptor (LXR) and FXR influence the activity of ChREBP; FXR is a key transcription factor of bile acid metabolism directly interacting with ChREBP. LXR alpha/beta double-knockout mouse studies conclusively showed reduced ChREBP activity and lipogenic gene expression in the liver. ChREBP inhibits the overactivation of cholesterol biosynthesis, thereby contributing to hepatoprotection in a high-fructose diet [127-129].

#### 8.1.2 Fat and Gene Expression

Dietary fat is one of the main macronutrients that aid in the development and growth of organisms. Besides being an energy source, dietary fat influences gene expression, resulting in cell differentiation and growth changes. Transcription factors regulated by fatty acids are identified in bacteria, amphibians, and mammals. These factors include peroxisome proliferator-activated receptors, Hepatocyte nuclear factor 4a (HNF4a), NFkB (nuclear factor kappa B), and SREBP1c (sterol regulatory element-binding protein 1c) [130]. These factors are regulated by directly binding fatty acids, fatty acyl-coenzyme A, or oxidized fatty acids. Eicosanoid regulation of G-protein-linked cell surface receptors, which activates signalling cascades, and oxidized fatty acid regulation of intracellular calcium levels that affects cell signalling cascades targeting the nucleus, also play a vital role in the regulation of these factors. The physiological reaction to fatty acids is dictated by the amount, chemistry, and duration of the fat consumed at the cellular level. It is also dependent on cell-specific fatty acid metabolism, the cellular abundance of specific nuclear and membrane receptors, and the involvement of specific transcription factors in gene expression. Polyunsaturated fatty acid (PUFA) suppresses the expression of SREBP-1 and the influence of SREBP-1 at the post-transcription level [130, 131]. PUFA can decrease the level of mature SREBP-1 protein, presumably increasing the intracellular regulatory pool of cholesterol. In LPDS media, depriving cells of cholesterol activates enzymatic cleavage, increasing mature SREBP-1 in the nucleus. In combination with increased mature SREBP-1, n-3 fatty acids (FA) did not reduce the leptin mRNA level. This reveals that the PUFA-induced effect on leptin expression may involve a reduction in mature SREBP-1. Studies have shown that PUFA decreases leptin gene expression *in vivo* and *in vitro* by reducing PPARg and SREBP-1 gene expression mechanisms [131, 132].

#### 8.1.3 Protein and Gene Expression

Recently, many studies have used omics technologies to determine the impact of protein intake on metabolism. Two hundred eighty-one genes were differentially regulated, at least two-fold in the liver of the protein-free group. Hat 11 genes in cholesterol metabolism were downregulated, and none of the genes in this pathway was up-regulated [133]. In the same study, liver transcriptomic profiles were also compared between two other diets: one made of gluten and the other based on casein; a lower number of differentially regulated genes was observed (61 upregulated and 50 downregulated in the gluten group) [133]. Like the comparison between protein-free and casein diets, cholesterol metabolism was highly sensitive to the source of proteins. All 15 differentially regulated genes in this pathway were unilaterally increased in the gluten group compared to the casein group. Interestingly, considering the opposite expression patterns of genes involved in hepatic cholesterol metabolism, both protein-free and gluten-free diets significantly decreased total serum cholesterol and HDL cholesterol [133-135].

#### 8.1.4 Mineral and Gene Expression

Minerals are involved in various gene expressions. Zinc, an essential trace element and cofactor, influences many metabolic pathways, hormone secretion pathways, and immune mechanisms [136]. One of the major components of ribonucleotide reductase and mitochondrial cytochromes is dietary iron. Iron deficiency results in decreased DNA repair efficiency and increases the chances of oxidative damage to mitochondrial DNA. The level of protein HFE (hemochromatosis) (a newly identified mutation in a major histocompatibility complex (MHC) class I-like gene) is increased by increasing the status of cell iron in human intestinal cells [137]. Magnesium is necessary for DNA polymerization, nucleotide excision repair, base excision repair, and mismatch repair, as well as microtubule polymerization and chromosome segregation [137].

#### 8.1.5 Vitamins and Gene Expression

Vitamins are essential components that are needed in both human and non-ruminant diets. Almost all vitamins present are involved in gene expression. Vitamin C and vitamin E help prevent DNA oxidation and lipid oxidation. At the same time, its deficiency is associated with increased baseline rates of DNA strand breaks, chromosome splits, oxidative DNA lesions, and lipid peroxide adducts on DNA. Folate and vitamin B12 play significant roles in the maintenance and methylation of DNA synthesis of dTMP from dUMP and efficient recycling of folate. Still, in a deficient condition, uracil misincorporation in DNA increases chromosome breaks, and DNA hypomethylation occurs [138]. Niacin is used as a substrate for poly (ADP-ribose) polymerase (PARP), which is involved in the cleavage and rejoining of DNA and the maintenance of telomere length. Its deficiency causes increased levels of unrepairable nicks in DNA, increased chromosome breaks and rearrangements, and sensitivity to mutagens. Vitamin A is involved in the gene expression of PEPCK (phosphoenolpyruvate kinase), IGF (insulin-like growth factor). Biotin is involved in various essential protein (enzymes) syntheses at the gene level. Vitamin C is involved in hepatic gene expression [138-140].

## 9 CRISPR-BASED TECHNOLOGIES AND THE FUTURE OF FOOD SCIENCE

“Genetic Engineering” refers to the artificial manipulation, modification, and recombination of DNA or other nucleic acid molecules in order to modify organisms or populations of organisms. CRISPR technology is an easy and efficient method for genome editing. Clustered Regularly Interspaced Short Palindromic Repeats (CRISPR) and CRISPR-associated sequences (Cas) are an adaptive immune system against invasive genetic elements in bacteria. It allows researchers to alter DNA sequences and modify gene functions easily. Correcting genetic abnormalities, treating and preventing the spread of diseases, and developing crops are only a few of its many possible applications [141, 142]. CRISPR has many practical advantages in the microbial industry, both natural and engineered, and could directly impact the future of food development. The CRISPR locus allows for strain typing, a method that fingerprints each strain, allowing for identifying proprietary blends in food fermentation and probiotic products. CRISPR naturally provides immunity against bacteriophages. However, this can be harnessed to vaccinate organisms with bacterial viruses widely encountered in large fermentations to decrease waste in the food-manufacturing process [143]. Finally, it is possible to implement CRISPR for the targeted killing of microbes. This may take the form of self-targeting, in which CRISPR acts as a programmable and specific antimicrobial agent. Self-targeting can be used as a selection marker in mixed bacterial populations to screen for unique alterations or unusual natural mutations. An antimicrobial agent would be used in mixed populations to remove undesirable organisms from culture blends. Bacterial genotyping is a widely used application of CRISPR, especially for industrially important *lactobacilli*, *Streptococci*, and foodborne pathogens, including *E. coli* and *Salmonella*. In these applications, researchers use the sequence variability within repeat-spacer arrays to distinguish strains. Strains can be completely identical in the rest of their genomes but still differences can be seen in the CRISPR systems. This allows companies to track their strains specifically [144].

## 10 SIGNIFICANCE OF GENOMICS IN FOOD SAFETY

Some years ago, laboratories at the FDA, USDA's food safety and Inspection Service, and the CDC relied almost exclusively on whole-genome sequencing (WGS) as their primary surveillance tool to differentiate bacteria strains and identify related clusters of infections. Moreover, 16S metagenomics techniques are used for screening the microbial flora in raw materials, production environment, and finished products. The US and many other countries use such molecular-based screening of imported food [145]. Whole-genome sequencing is the technique of determining the complete DNA sequence of an organism's genome, which is the organism's total genetic content. The total genetic content includes chromosomal DNA, mitochondria, and chloroplast (for plants). WGS is used for identifying and screening food such as meat. More advancements in the future will bring many food items into this list. Genetic material from the species is used as markers (molecular signatures) for identifying the material. Establishing these markers has become easy with WGS. This can help agencies to reduce adulterations and fraudulence in food [146]. The 16S ribosomal RNA (rRNA) sequences that are relatively short are robust markers among all available markers for identifying various species of bacteria as they are often conserved within a species and generally different between species. The advancement of next-generation sequencing, coupled with the improvement in the lab protocols for capturing 16S rRNA, has made the process of sequencing very easy, fast, and cheap. This technique is termed 16S-metagenomics or metagenomics and has been used to identify bacterial species in any biological sample environment. Unlike the traditional methods, this technique captures the entire flora, and it can identify novel organisms and hard-to-culture species from the samples. As this technique targets the 16S rRNA, it is easy to separate the microbial material from meat or vegetable samples [142].

### 10.1 Metagenomics for Safety Measures, Improved Sanitation, and Better Shelf Life

As metagenomics has become very feasible in terms of cost and time while offering higher accuracy, food processing industries have started using it as a tool for safety auditing and quality measures. It enables them to check the microbiome flora in their raw materials, production environment, storage, *etc*. Hence, it helps modify the sanitation protocols based on the microbiome with newly identified food spoilage organisms. Screening using metagenomics allows us to understand how microbes behave within the processing environment and spoiled food. The food plant's microbiome, with details from all plant sites, makes it easy to identify spots and the process that requires improvement [147, 148]. Metagenome and WGS are coupled together to identify and prevent foodborne diseases while assisting us in tracing back to the source where it originated (maybe a supply chain, production line, or raw material) [149]. WGS facilitates the highly sensitive “precision” subtyping that assists the detection, with higher accuracy, of foodborne disease outbreaks and aids the comprehensive characterization of foodborne pathogens and identifying strains and clonal groups that differ in virulence and antimicrobial resistance [150, 151].

### 10.2 Metagenome Sequencing and Data Analysis

**Fig. (5):**
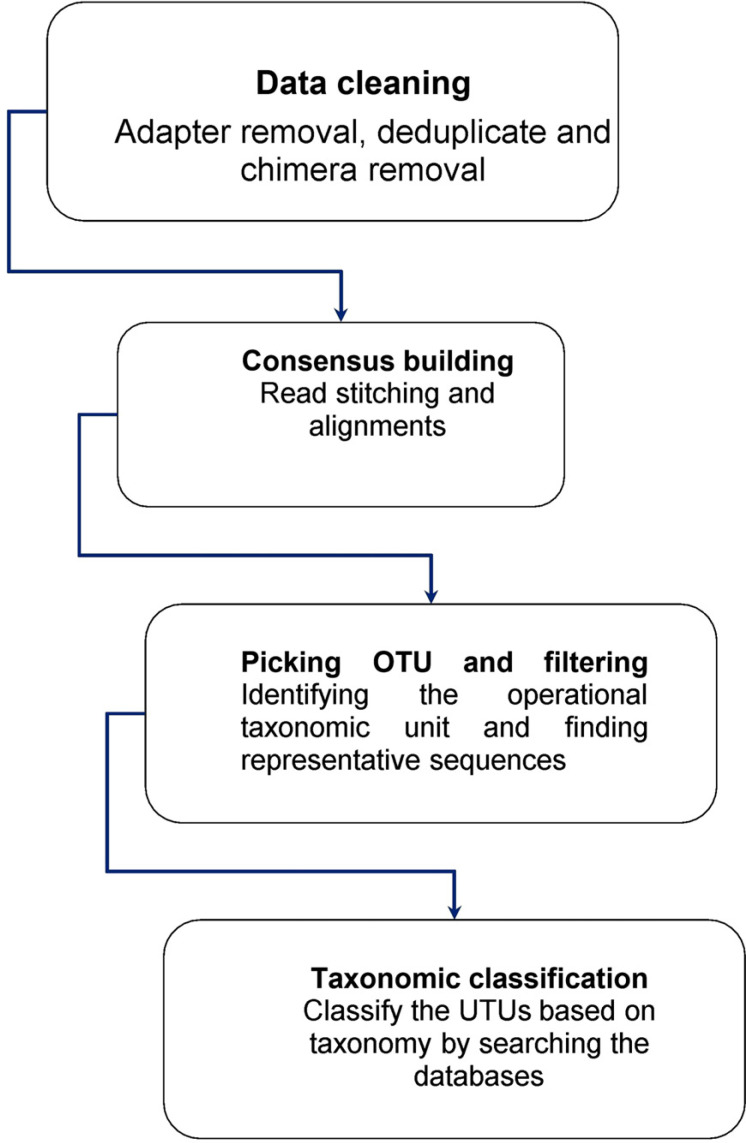
Overview of the process of metagenome data analysis.

The study of a set of genetic material (genomes) from diverse organisms is known as metagenomics. The term “metagenomics” is generally used to describe the study of microbial communities to provide details on a given environment's microbial diversity and ecology (Fig. **5**) [152]. The steps for metagenome sequencing and data analysis are shown in Table **7**.

## 11 WHOLE-GENOME SEQUENCING AND DATA ANALYSIS

Molecular profiling technologies are highly accessible and can be a potential tool to understand drug-food interactions with better resolution. Emerging technologies that can profile genome (genomics), transcriptome (transcriptomics), microbiome (microbiomics), metabolome (metabolomics), epigenome (epigenomics), *etc*., can also be combined and analyzed in a multi-omics framework to understand the molecules and potential interactions driving drug-food interactions. In the following section, we briefly discuss the concepts in genomics that are relevant to studying food-drug interactions.

### 11.1 Short-read

Short-read sequencing is the best platform for generating high-quality deep coverage for small to large size genomes. However, short read lengths have limitations in resolving complex regions, especially those with repetitive or heterozygous sequences. Popular systems currently available produce short reads of size 50 to 150 base pairs (bp). Generated raw data will be in fastq format, and the tranche of it is shown in Fig. (**6**). It usually generates millions of short reads for one organism of study [153].

### 11.2 Long-read

Recent advancements have made a big leap from the short-read technology and invented a few different methodologies to read much longer DNA fragments at the cost of a minimal quality drop. Currently, there are a few platforms that read DNA fragments of sizes from 10,000 bp (10 kbp) to 60,000 bp (60 kbp). Data quality compromises are found to be increasing proportionately with the read length [153, 154].

### 11.3 Linked-read

Linked-Reads provide long-range information from short-read sequencing data, which enables the capture of information that would have been missed with other methods. Linked-reads utilizes molecular barcodes to tag reads from the same long DNA fragment. These unique barcodes added to every short read generated from an individual molecule enable one to link the short reads together and arrange them into pseudomolecules (pseudo chromosomes). Chromosome conformation capture (3C/Hi-C/Capture-C) method is used to study the 3-D architecture of genomes, which helps unveil the 3-D folding of chromosomes and the arrangement of distant functional elements, such as promoters and enhancers, and hence reveals the relationship between chromosome organization and genome activity. Hi-C methodology produces high-quality short-read data that help arrange the assembled contigs/scaffolds for building chromosome-level assemblies [155].

### 11.4 Optical Mapping

Optical mapping platforms use optical maps generated by fluorescent-labelled ultralong DNA molecules of >150 kb in length. A de novo optical map assembly can be compared to a DNA sequence assembly. It can be used to correct wrong assemblies, stitch scaffolds, and correct incorrect assembly of contigs sequences in highly complex areas of the genome. This platform's next-generation mapping combines proprietary Nano-channel arrays with optical mapping to image extremely long, high-molecular-weight DNA in its most native state. Optical mapping also enables structural variation detection with high sensitivity and obtains highly contiguous genome assemblies [156, 157]. The data generated can be analyzed mainly in two different strategies based on the availability of the reference genome. In that case, the study will focus on the variations in the genome of the individual (variant calling analysis mentioned below) while the target would be to build a genome assembly (de novo analysis discussed below) if the genome is not available.

### 11.5 *De novo* Assembly and Annotation and Genome Assembly

Genome assembly stitches millions of DNA fragments together to form the DNA sequences. All sequencing protocols (short and long read) generate multiple copies of overlapping fragments. The assembly algorithms search for the overlapping pieces and stitch them to build bigger pieces as long as possible. The overall process of genome assembly building is illustrated in Fig. (**7**) [158]. Genome assembly is a computationally challenging task that requires a high amount of memory (RAM) and storage (hard disk space). Computational and time requirement is exponentially proportional to the genome size and input data size. Many different algorithms are available in the open-source domain for building assemblies from various data types. Many of them are hybrid assemblers capable of using different data types together and building the assembly. Extensive research is focused on this, and better strategies are coming out every year [159, 160]. Popular assembly tools are listed in Table **8**.

### 11.6 Repeat Discovery

Almost all eukaryotic and few prokaryotic genomes contain different repeat elements associated with many structural (*e.g*., chromosomal organization) and functional (*e.g*., gene regulation) activities. There are established strategies to discover repeat regions and repeats that use models with priori knowledge. RepeatMasker is one such tool. This strategy fails to identify novel repeats, and some species have large numbers of novel repeats. A couple of computational tools (*e.g*., RepeatModeler) are currently proven to perform the task of novel repeat identification [161,162].

### 11.7 Gene Discovery

Genes are functional elements of genomes. Identifying the gene components in the genome is termed gene discovery. The task is to determine the coordinates of such components in the assembled genome. *Ab initio* strategies are utilized heavily in this process, where the mathematical models built using the priori knowledge are utilized as supporting data. Some of these algorithms allow us to use custom-made models too. Some tools accept protein sequences, RNA sequences, and sequences of expressed sequence tags (EST) and use them as evidence to score the ab initio predictions [163, 164].

#### 11.7.1 Function Annotation of Genes

**Fig. (8):**
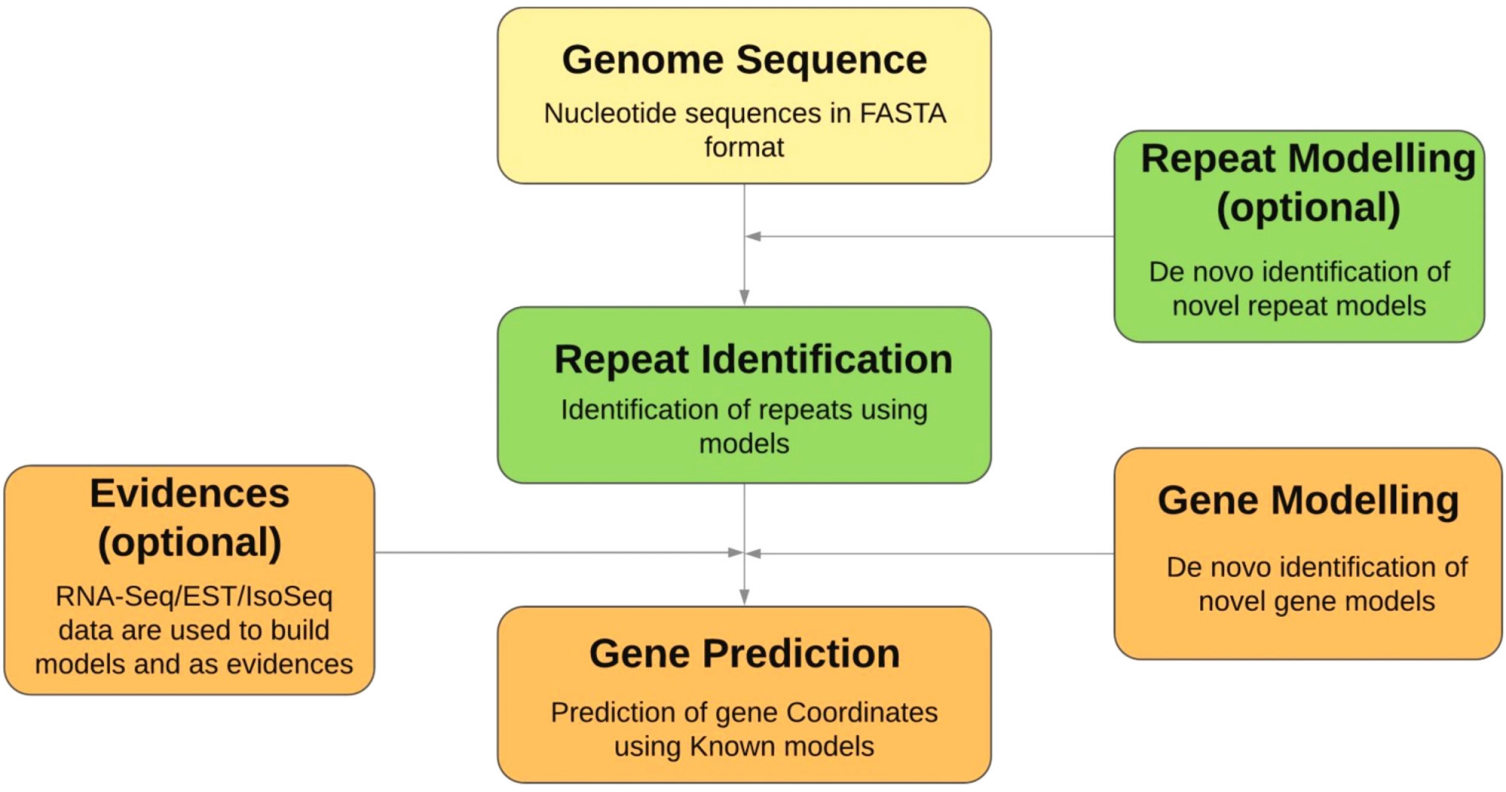
Process of genome annotation in detail.

Identifying the functions of the genes discovered are called function annotation. The most popular strategy for this task is to sequence similarity-based search (homology search) in various sequence databases like NCBI and UniProt. Multiple blast algorithms are used in this process. Computational tools like “Prediction of Unassigned Regions: PURE; available *via* https://caps.ncbs.res.in/PURE, can be used to assign functions to novel protein regions. Researchers around the globe use highly customized strategies that utilize knowledge related to protein domains, protein family, ortholog clusters, *etc*. (Fig. **8**) [165-169].

## 12 EMERGING TRENDS IN DRUG-FOOD INTERACTIONS AND OUTLOOK

Drug-nutrition interaction at present is an area that is highly underrated and overlooked. Adult multimorbidity is increasing rapidly, contributing to polypharmacy and raising the likelihood of significant drug-drug interactions. Vitamins as essential nutrients have central metabolism functions; therefore, interactions and insufficient availability of vitamins can lead to critical metabolism impairments. The effect of a patient's diet or dietary supplements on medication and drug effectiveness is an emerging area. Especially in the field of oncology, there is a growing trend towards oral delivery of anticancer drugs and self-medication with nutritional supplements; thus, the knowledge of the kinetics and chemistry of the activation of anticancer bio-reductive medicines, identifying the metabolic enzymes involved in the bioactivation, and data of their nutritional regulation will help in managing cytotoxicity.

Developing new functional foods to optimize the interaction between current foods and current drugs and identify novel functionalities of both old and new food ingredients while addressing multiple aspects of the patient's nutritional challenges are emerging areas filled with potential and promise. A better understanding of drug-food interactions offers the possibility of setting specific dietary guidelines and recommending special diets, suitable foods, or supplements to patients, thereby increasing the effectiveness of the treatment.

## 13 CURRENT AND FUTURE DEVELOPMENTS

Dietary risks impact people of all ages, genders, and sociodemographic backgrounds. Food can be used to avoid chronic diseases and metabolic syndromes, like obesity, diabetes, hypertension, dyslipidemia, and drug-nutrient interactions could alter the pharmacokinetics and pharmacodynamics of drugs. Pharmacokinetics is the quantitative elucidation of drug disposition that consists of absorption, metabolism, distribution, and excretion, whereas pharmacodynamics is the clinical effect of the drug, which can be manifested either by a reduction in bioavailability or an increase in bioavailability that can result in toxicities [55, 57, 76].

The ability of food to interfere with drug treatment is a major concern in clinical practice. Knowledge of drug-food interactions is helping us identify, predict, and prevent unwanted interactions between food and marketed or novel drugs. To interpret the biological responses to diet, as well as to assign the importance of drug-food interaction, all available information on the chemical background of the small molecules in our diet is assembled on interaction networks [71, 74].

Nutrigenetics and nutrigenomics help to map the genome's effects and genetic variations on food/nutrients intake and its role in gene expression. High-throughput omics technologies and genomic information allow us to understand the nutrient-gene interaction and help develop personalized nutrition [118, 170, 171]. Whole-genome sequencing helps determine the complete DNA sequence of an organism’s total genetic content or its genome. Nutrigenetics and nutrigenomics are becoming ever more evident and play a central role in analysing dietary impacts on health outcomes and the systematic assessment of nutrient impacts by a multitude of 'omic' technology biomarkers. Some of these technologies are still in their infancy, while others are much more mature [145, 172]. As metagenomics has become very feasible in terms of cost and time while offering higher accuracy, food processing industries have started using it as a tool for safety auditing and quality measures. It enables them to check the microbiome flora in their raw materials, production environment, storage, *etc*. Hence, it helps modify the sanitation protocols based on the microbiome with newly identified food spoilage organisms. Metagenome and WGS are coupled together to identify and prevent foodborne diseases while assisting us in tracing back to the source of contamination.WGS facilitates the highly sensitive “precision” subtyping, which assists the detection, with higher accuracy, of foodborne disease outbreaks and aids the comprehensive characterization of foodborne pathogens and identification of strains and clonal groups that differ in virulence and antimicrobial resistance [141, 173].

## CONCLUSION

Drug-food interaction holds utmost importance in the era of personalised health. The pharmacological effect of food on drugs has been acknowledged since time immemorial. Nevertheless, research and database of drug-food interaction are dire compared to the drug-drug interaction and drug-protein interaction. In summary, the review has shed light on drug-food interaction's mechanism, types and how this knowledge can be manipulated by leveraging artificial intelligence, machine learning, next-generation sequencing and CRISPR for a better clinical outcome.

## Figures and Tables

**Fig. (6):**
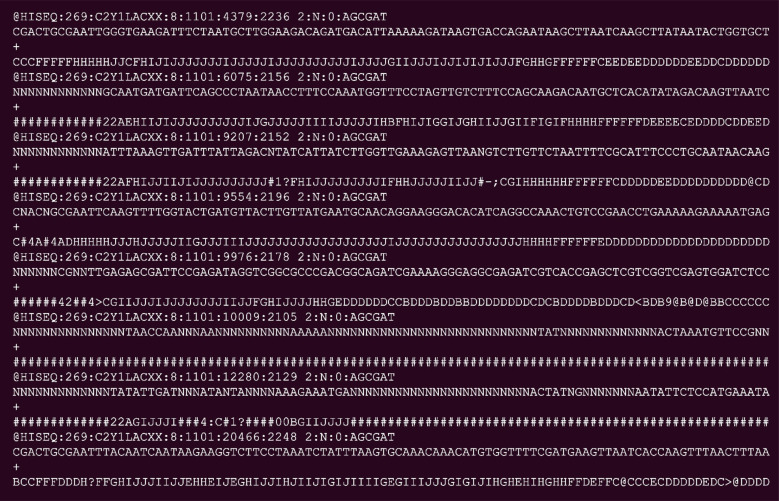
Tranche of short-read sequence output.

**Fig. (7):**
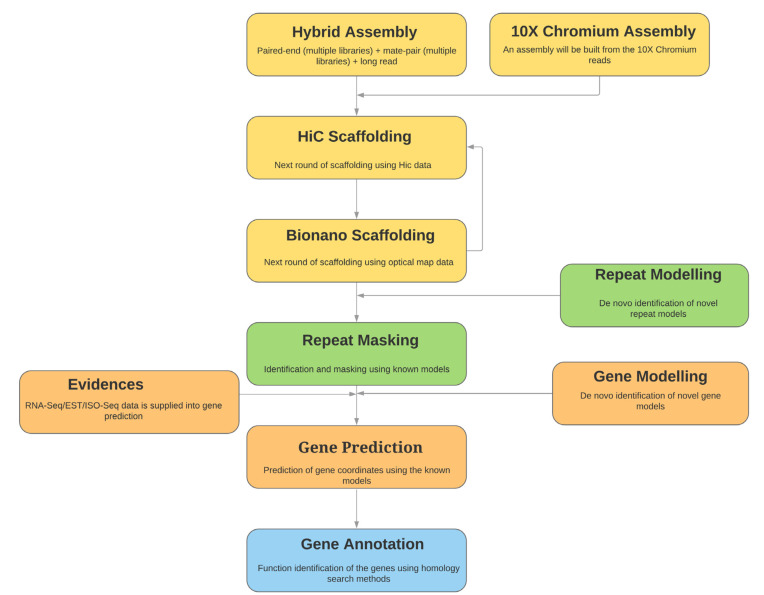
Overview of the process of the genome assembly building.

**Table 1:** Functional foods and their protective cardiovascular properties.

**Functional Food**	**Bioactive Compounds**	**Mode of Action**
Nuts	Tocopherols	α-tocopherol inhibits the oxidation of low-density lipoprotein cholesterol. α-tocopherol shows anti-inflammatory activity and modulates the expression of proteins involved in the uptake, transport, and degradation of atherogenic lipids [18].
Soy proteins	Genistein and daidzein	Reverse stress-induced alteration of protein expression profile in postmenopausal women [19].
Extravirgin olive oil	Polyphenolics and oleic acid	Improve anti-oxidant capacity, mitochondrial function, and autophagy while reducing oxidative stress, inflammation, and cellular senescence in vascular smooth muscle cells (VSMCs) and endothelial cells (ECs) [20].
Citrus fruits	Ascorbic acid	Vitamin C intake is inversely associated with cardiovascular mortality [21].
Grapes and red wines	Anthocyanins, catechins, cyanidings, flavonols, myricetin, and quercetin	Prevent cardiovascular health problems through bio-efficacy on vascular parameters, myocardial conditions, and metabolism [22].

**Table 2:** Functional food’s role in cancer prevention.

**Bioactive Compound**	**Functional Food**	**Medicinal Properties**
Phyto-estrogens	Soya foods	Anti-tumor [37]
Flavonoids	Plants	Anti-oxidant [38]
Omega-3	Fish	Anti-inflammatory [39]
Fiber	Vegetable and cereals	Antitumor [40]
Isothiocyanates	Broccoli, cauliflower, kale	Anti-tumor [41]
Astaxanthin	Green algae, salmon	Antioxidant [42]
Fucoxanthin	Brown algae, heterokonts	Anti-tumor [43]
Lutein	Dark green leafy vegetables	Anti-tumor [44]

**Table 3:** Fruits-drug interactions.

**Fruit**	**Phytochemicals**	**Drug Interactions**
Apple	Phenolic acids (tannins), flavonoids (including quercetin), glycosylated xanthones (mangiferin), and saponins	Fexofenadine [61].
Cranberry	Flavonoids, such as anthocyanidin (cyaniding and poenidin), and flavonols (quercetin) and carotenoids	Warfarin, *In vitro* system Diclofenac [61, 62].
Grapefruit	Bergamottin, flavonoids (nobilein, tangeretin, quercetin, diosmin, naringenin, naringin, and kaempferol), and furanocoumarins	Calcium channel antagonist,central nervous system modulators, HMG-CoA reductase, immunosuppressants, antibiotics [61, 63].
Mango	Phenolic acids (tannins), flavonoids (anthocyanins), carotenoids, essential oils, fatty acids, lectins, phenols, saponins, alkaloids, and triterpenes	Midazolam, diclofenac,chlorzoxazone, verapamil [61, 64].
Pomegranate	Phenolic acids (punicalagin and tannins), flavonoids (anthocyanins), and pectin	Carbamazepine [61, 65].
Seville orange	Naringin, limonin, flavonoids, hesperidin, limonene, carotenoids, iso hesperidin, terpineol	Vinblastine, fexofenadine,ciprofloxacin, ciclosporine [61, 66].
Tangerine	Flavonoids as diosmin, tangeritin, nobilein, and quercetin	Nifedipine, Digoxina [61, 62, 67].

**Table 4:** Vegetable-drug interactions.

**Vegetables**	**Phytochemicals**	**Drug Interactions**
Spinach	Flavonoids and *p* ‐coumaric acid derivatives, α‐lipoic acid, polyphenols, lutein, zeaxanthin, betaine	*In vitro* system: heterocyclic aromatic amines [68].
Tomato	Carotenoids phytofluene, phytoene, neurosporene, γ‐carotene, and ζ‐carotene lycopene, phytoene, phytofluene, quercetin, polyphenols, kaempferol	Diethylnitrosamine, N-methyl-N-nitrosourea and 1,2 dimethylhydrazine [69].
Watercress	Phenylethyl isothiocyanate (PEITC) and methylsulphinyl alkyl isothiocyanates (MEITCs), flavonoids such as quercetin, hydroxycinnamic acids, and carotenoids such as β‐carotene and lutein	Chlorzoxazone [70].

**Table 5:** Food-drug interactions.

**Drugs**	**Food**	**Mechanism**
ACE Inhibitors	Empty stomach	Absorption is increased [75-78].
Acetaminophen	Pectin	Delays absorption [75-78].
Celiprolol	Orange juice	The intestinal absorption is inhibited [75-78].
Cycloserine	High fat meals	Decreases the serum concentration [75-78].
Esomeprazole	High-fat meal	Decreases bioavailability [75-78].
Fexofenadine	With grapefruit juice	Reduces absorption [75-78].
Glimepiride	with breakfast	Absolute bioavailability [75-78].
Lanoxin	With licorice	Increases the risk of drug toxicity [75-78].
NSAIDS	Alcohol	Can increase the risk of liver damage or stomach bleeding [75-79].
Penicillin	Empty stomach	Absorption is increased [75-78].
Tricyclics	High protein diet, Vitamin C rich food	Reduce absorption of drugs [75-78]
Warfarin	High-protein diet Vegetables containing vitamin k	Raise serum albumin levels and decrease the international normalized ratio (INR). Interfere with the effectiveness and safety of warfarin therapy. Decrease warfarin activity. Elevated INR without bleeding in an elderly patient. Thromboembolic complications may develop, and hypoprothrombinemic effect of warfarin may be decreased [75-78, 80]

**Table 6:** Types of nutrient-gene interactions.

**S. No.**	**Type of Interactions**	**Example**
1	Direct interactions In direct interaction upon contact with the receptor, the nutrients act as transcription factors that bind to DNA and induce gene expression.	Vitamin A/vitamin A derivatives interact with receptor proteins of retinoic acid and activate or repress transcription [119, 120].
2	Epigenetic interactions In Epigenetic interaction, the gene expression is affected chronically as major alteration happens to the structure of DNA.	Epigenetic modifications, such as methylation, acetylation, or biotinylation of histones lead to significant gene expression variations that can persist throughout the person's life. [119, 120].
3	Genetic variation The expression and functionality of genes are altered due to genetic variations, such as single-nucleotide polymorphisms (SNPs).	The PEMT gene codes for a protein responsible for endogenous choline production in the liver. An SNP in the promoter region of the PEMT gene (rs12325817) is associated with choline deficiency in humans with significantly increased susceptibility to choline deficiency in women [119, 120].

**Table 7:** Steps for metagenome sequencing and data analysis.

**Step. No.**	**Name**	**Details**
Step 1	Data clean up	Sequencing adapters and duplicated reads will be removed to clean up the data. Sequence chimeras are an artifact of PCR amplification and subsequent next-generation sequencing. Chimeras are removed using *de novo* chimera removal tools, like UCHIME of VSEARCH or a reference-based strategy.
Step 2	Consensus building	The forward primer (V3 specific) and reverse primers (V4 specific) are trimmed, and the reads with a quality threshold (minimum Phred score of 20) that have both pairs are identified and used for the generation of V3-V4 consensus. V3-V4 consensus amplicons are built using specialized tools like FLASH by merging these reads into an overlapping region with a certain quality length.
Step 3	Picking OTU and filtering	Total reads from all samples are pooled and clustered into OTUs based on their sequence similarity, using specialized tools like Uclust, with certain similarity scores. The representative sequences from each clustered OTUs are picked and aligned against curated knowledge bases, like SILVA, using tools like PyNAST.
Step 4	Taxonomic classification	Taxonomy classification can be performed using the RDP classifier by mapping each representative sequence against the SILVA OTUs database.

**Table 8:** Popular assembly tools.

**Assembly Tool**	**Refs.**	**Type of Input Data**
Velvet	Zerbino, D. R., & Birney, E. (2008). Velvet: Algorithms for de novo short read assembly using de Bruijn graph. Genome Research, 18(5), 821-829. https://doi.org/10.1101/gr.074492.107	Illumina PE, Illumina MP
ABySS	Simpson, J. T., Wong, K., Jackman, S. D., Schein, J. E., Jones, S. J. M., & Birol, I. (2009). ABySS: A parallel assembler for short-read sequence data. Genome Research, 19(6), 1117-1123. https://doi.org/10.1101/gr.089532.108	Illumina PE, Illumina MP
SPAdes	Anton Bankevich, Sergey Nurk, Dmitry Antipov, Alexey A. Gurevich, Mikhail Dvorkin, Alexander S. Kulikov, Valery M. Lesin, Sergey I. Nikolenko, Son Pham, Andrey D. Prjibelski, Alexey V. Pyshkin, Alexander V. Sirotkin, Nikolay Vyahhi, Glenn Tesler, Max A. Alekseyev, and Pavel A. Pevzner. SPAdes: A New Genome Assembly Algorithm and Its Applications to Single-Cell Sequencing. Journal of Computational Biology 19(5) (2012), 455-477	Illumina PE, Illumina MP, PacBio, Nanopore
Unicycler	Wick, R. R., Judd, L. M., Gorrie, C. L., & Holt, K. E. (2017). Unicycler: Resolving bacterial genome assemblies from short and long sequencing reads. PLoS Computational Biology, 13(6), 1-22. https://doi.org/10.1371/journal.pcbi.1005595	Illumina PE, Nanopore, PacBio
SOAPdenovo2	1. Luo, R., Liu, B., Xie, Y., Li, Z., Huang, W., Yuan, J. & Wang, J. (2012). SOAPdenovo2: an empirically improved memory-efficient short-read de novo assembler. Gigascience, 1(1), 18.	Illumina PE, Illumina MP
MaSuRCA	Zimin, A. V., Marçais, G., Puiu, D., Roberts, M., Salzberg, S. L., & Yorke, J. A. (2013). The MaSuRCA genome assembler. Bioinformatics, 29(21), 2669-2677	Illumina PE, Illumina MP, PacBio, Nanopore
Redundans	1. Pryszcz, L. P., & Gabaldón, T. (2016). Redundans: An assembly pipeline for highly heterozygous genomes. Nucleic Acids Research, 44(12), e113. https://doi.org/10.1093/nar/gkw294	Illumina PE, Illumina MP
flye	Lin, Y., Yuan, J., Kolmogorov, M., Shen, M. W., Chaisson, M., & Pevzner, P. A. (2016). Assembly of long error-prone reads using de Bruijn graph. https://doi.org/10.1073/pnas.1604560113/-/DCSupplemental.www.pnas.org/cgi/doi/10.1073/pnas.1604560113	PacBio, Oxford Nanopore
canu	Koren, S., Walenz, B. P., Berlin, K., Miller, J. R., Bergman, N. H., & Phillippy, A. M. (2017). Canu: scalable and accurate long-read assembly *via* adaptive k -mer weighting and repeat separation. 722-736. https://doi.org/10.1101/gr.215087.116.Freely	PacBio, Oxford Nanopore
Platanus	Kajitani, R., Toshimoto, K., Noguchi, H., Toyoda, A., Ogura, Y., Okuno, M., … Itoh, T. (2014). Efficient de novo assembly of highly heterozygous genomes from whole-genome shotgun short reads. 1384-1395. https://doi.org/10.1101/gr.170720.113.Freely	Illumina PE, Illumina MP
ALLPATHS-LG	Gnerre, S., Maccallum, I., Przybylski, D., Ribeiro, F. J., Burton, J. N., Walker, B. J., … Jaffe, D. B. (2011). High-quality draft assemblies of mammalian genomes from massively parallel sequence data. 108(4), 1513-1518. https://doi.org/10.1073/pnas.1017351108	Illumina PE, Illumina MP, PacBio
FALCON	Kronenberg, Zev & Hall, Richard & Hiendleder, Stefan & Smith, Tim & Sullivan, Shawn & Williams, John & Kingan, Sarah. (2018). FALCON-Phase: Integrating PacBio and Hi-C data for phased diploid genomes. 10.1101/327064.	PacBio long reads
SMARTdenovo	Hailin Liu,Shigang Wu,Alun Li,Jue Ruan,SMARTdenovo: a de novo assembler using long noisy reads,Gigabyte,1,2021 https://doi.org/10.46471/gigabyte.15	PacBio, Nanopore
Miniasm	Li, H. (2016). Sequence analysis Minimap and miniasm: fast mapping and de novo assembly for noisy long sequences. 32(March), 2103-2110. https://doi.org/10.1093/bioinformatics/btw152	PacBio, Nanopore
LRscarf	Qin, M., Wu, S., Li, A., Zhao, F., Feng, H., Ding, L., & Ruan, J. (2019). LRScaf: improving draft genomes using long noisy reads. 1-12.	PacBio, Nanopore

## LIST OF ABBREVIATIONS

ARE: Antioxidant Response Element
IBD: Inflammatory Bowel Disease
INR: International Normalized Ratio
RNA: Ribonucleic Acid
EST: Expressed Sequence Tags
